# Imaging the Visual Network in the Migraine Spectrum

**DOI:** 10.3389/fneur.2019.01325

**Published:** 2019-12-13

**Authors:** Francesca Puledda, Dominic Ffytche, Owen O'Daly, Peter J. Goadsby

**Affiliations:** ^1^Headache Group, Department of Basic and Clinical Neuroscience, Institute of Psychiatry, Psychology and Neuroscience, King's College London, London, United Kingdom; ^2^NIHR-Wellcome Trust King's Clinical Research Facility, SLaM NIHR Biomedical Research Centre, King's College Hospital, London, United Kingdom; ^3^Department of Old Age Psychiatry, Institute of Psychiatry, Psychology and Neuroscience, King's College London, London, United Kingdom; ^4^Department of Neuroimaging, Centre for Neuroimaging Sciences, Institute of Psychiatry, Psychology and Neuroscience, King's College London, London, United Kingdom

**Keywords:** migraine, migraine spectrum, neuroimaging, visual snow, visual network, aura, photophobia

## Abstract

The involvement of the visual network in migraine pathophysiology has been well-known for more than a century. Not only is the aura phenomenon linked to cortical alterations primarily localized in the visual cortex; but also migraine without aura has shown distinct dysfunction of visual processing in several studies in the past. Further, the study of photophobia, a hallmark migraine symptom, has allowed unraveling of distinct connections that link retinal pathways to the trigeminovascular system. Finally, visual snow, a recently recognized neurological disorder characterized by a continuous visual disturbance, is highly comorbid with migraine and possibly shares with it some common pathophysiological mechanisms. Here, we review the most relevant neuroimaging literature to date, considering studies that have either attempted to investigate the visual network or have indirectly shown visual processing dysfunctions in migraine. We do this by taking into account the broader spectrum of migrainous biology, thus analyzing migraine both with and without aura, focusing on light sensitivity as the most relevant visual symptom in migraine, and finally analyzing the visual snow syndrome. We also present possible hypotheses on the underlying pathophysiology of visual snow, for which very little is currently known.

## Key concepts

- A key feature of migraine, during and between attacks, is represented by altered visual cortex excitability. Multiple functional and structural neuroimaging studies have shown alterations in several areas of the visual network in migraine both with and without aura compared to controls, particularly of the motion processing network.
- Visual symptoms are the most common clinical manifestation of the aura phenomenon. The neurophysiological correlate of visual aura is likely represented by cortical spreading depression, starting in the extrastriate area V3A. Neuroimaging has shown in various forms that migraine with aura is characterized by lower and higher visual processing impairment, both ictally and interictally.
- Photophobia is an important aspect of migraine biology, present during, before and after the headache attacks. Migrainous photophobia is most likely linked to abnormal sensory processing in thalamic structures, particularly the pulvinar.
- Visual snow is a neurological disorder, commonly comorbid with migraine, characterized by a continuous visual disturbance that takes the form of uncountable tiny flickering dots covering the entire visual field. Its underlying pathophysiology is possibly characterized by a combination of peripheral, subcortical, and cortical dysfunctions causing an increased perception of normally subthreshold visual stimuli.

## Introduction

In the last decades, imaging has gained considerable interest in the field of neuroscience and has allowed researchers to begin to unravel important mechanisms in the biology of complex neurological disorders. Several conventional and more advanced neuroimaging techniques have been implemented over the years and have proven to be important tools in the understanding of normal and pathological brain biology.

In the field of primary headaches, and migraine in particular, a growing body of neuroimaging work has served the purpose of dissecting important structural and functional alterations that characterize the disorder. One of the main aspects that has emerged from these studies is the confirmation, previously shown through animal models, that migraine does not represent a primary vascular disorder, rather a complex brain dysfunction involving several cortical and subcortical networks (1, 2).

The visual network has been one of the most studied systems in the migraine brain for several reasons. The most obvious explanation is certainly linked to the intriguing phenomenon of aura, a fully reversible neurological dysfunction which occurs in about a third of migraine cases and is represented chiefly by positive or negative visual symptoms (3, 4).

Another reason for the rising interest in studying visual function has been photophobia, a clinical hallmark of migraine both during attacks and in the interictal phase (5, 6). Recent evidence has led to better insight on the link between light inputs and pain, through the discovery of a pathway where photic signals from the retina converge on thalamic trigeminovascular neurons (7).

Finally, the notion that visual function is abnormal in migraineurs even in between attacks has lead researchers in the past to carry out extensive neurophysiological investigation of the visual network in migraine (8, 9). This uncovered important pathophysiological mechanisms now known to be typical of the migrainous brain, such as lack of habituation (10, 11). This particular form of altered excitability has been found interictally (12), although it typically fluctuates through the migraine cycle and can revert with disease chronification (13).

The visual brain is an extremely complex system consisting of multiple, hierarchical nodes which specialize in different functions at different times. These separate systems—which are incredibly uniform at a cytoarchitectonic level within the human cortex—work in parallel synchrony and autonomously from each other, resulting in the final conscious percept of vision (14). The complex integration between different areas of the visual network is made possible by existing connections between different cortical and subcortical areas specializing in different aspects of vision, and also between other cortical sensory, attentional, and cognitive processing networks (15, 16). The visual motion network is a perfect example of such integration and hierarchical sub-specialization, and it is particularly relevant in migraine biology, as this review will highlight. The motion network is composed chiefly of visual area V5, which specifically responds to motion stimuli, of sub-compartments within V1/V2, of area V3/V3A in the cuneus and finally of Brodmann area (BA) 7 in the precuneus (17).

In this review, we focus on neuroimaging findings that have shown direct involvement of the visual network in migraine. We will review studies broadly considered as being in the “migraine spectrum,” thus focusing on migraine both with and without aura, photophobia, and finally visual snow.

Visual snow (VS) is a common comorbidity of migraine, with which it may share some pathophysiological mechanisms (18, 19). In addition to describing the limited neuroimaging literature available for VS, we will proceed to present distinct hypotheses for putative pathophysiological mechanisms underlying visual snow, hoping to elucidate the neurobiology of the disorder and provide insight for future studies attempting its investigation.

## Methods

For the purpose of this narrative review, we performed a literature search using PubMed database in April 2019, with the following key words: “migraine,” “aura,” “migraine with aura,” “migraine without aura,” “visual snow,” “prolonged aura,” “visual,” “visual network” combined with “imaging,” “neuroimaging,” “BOLD,” “functional MRI,” “fMRI,” “VBM,” “PET,” “spectroscopy.” Articles were chosen based on their relevance to the topic. The reference lists of most publications and any other relevant papers known to the authors were further reviewed.

## The Visual Network in Migraine Biology

In the last decades, we have learnt much about migraine pathophysiology by studying the visual system of migraineurs, particularly in, but not limited to, the context of aura and light hypersensitivity. Both structural and functional neuroimaging techniques have been used for this purpose. The majority of studies have focused on the interictal migraine phase, as this is generally more practical, however, an increasing number of recent studies have also successfully investigated the ictal phase. This has been achieved either by imaging attacks of spontaneous onset (20) or by triggering headache through different forms of pharmacological provocation (21).

Functional imaging approaches are particularly suitable for a disorder characterized by pathological network dysfunction such as migraine. Positron emission tomography (PET) -using different radiotracers to investigate brain metabolism—and functional magnetic resonance imaging (fMRI), either with visual stimuli to capture the blood-oxygen-level-dependent (BOLD) responses or scanning during the resting state to study brain connectivity, are commonly used techniques in this context. These powerful approaches have uncovered important information regarding brain function and network configuration between attacks and at their initiation.

Structural techniques, such as voxel and surface-based morphometry (SBM) or DTI, on the other hand, provide insights on the morphological characteristics of key gray and white matter structures that are implicated in the biology of the migrainous brain.

Finally, magnetic resonance spectroscopy (MRS) allows to investigate brain metabolism directly.

### Functional Neuroimaging in Migraine

Several functional neuroimaging studies have shown a dysfunction of the visual network in migraine, both with (MwA) and without aura (MwoA). A summary of these is provided in Table 1.

**Table 1:** Main functional neuroimaging studies investigating the visual network in migraine with (MwA) and without aura (MwoA).

**References**	**Patient cohort**	**Migraine phase and attack type**	**Methodology**	**Main results**
Hadjikhani et al. (22)	2 MwA	Ictal and during aura; 2 spontaneous and 3 induced attacks with physical exercise	Event-related fMRI with visual stimulus (checkerboard on/off pattern every 16 s)	Focal increase followed by a decrease in BOLD signal, starting in area V3A of extrastriate cortex (lingual gyrus), and progressing congruently with retinotopic representation of visual aura percept
Cao et al. (23)	10 MwA 2 MwoA	Ictal and during aura (in 4 MwA patients); induced attacks with visual stimulation	Event-related fMRI with visual stimulus (checkerboard on/off pattern every 14 s)	Visual stimulus can trigger migraine attacks (with and without aura) through the activation of brainstem structures (red nucleus and substantia nigra)
Vincent et al. (24)	5 MwA	Interictal	Event-related fMRI with visual stimulus (alternating lines simulating zigzags of aura)	Increased activation of extrastriate cortex respect to controls
Boulloche et al. (6)	4 MwA, 3 MwoA, episodic	Interictal	${\text{H}}_{2}^{15}$O PET with visual stimulus (luminous stimulation at three intensities) with and without noxious trigeminal heat stimulation	Light stimulation caused increased striate and extrastriate visual cortex activation (cuneus, lingual gyrus, and posterior cingulate cortex) in migraineurs respect to controls
Antal et al. (25)	12 MwA 12 MwoA	Interictal	Event-related fMRI with visual motion stimulus (moving dots alternated with static dots)	Decreased activation of inferior-posterior V5 complex (middle temporal area) and increased activation of superior-anterior V5 complex in migraineurs respect to controls, showing that higher-order visual areas are affected in migraine
Martin et al. (26)	7 MwA 12 MwoA	Interictal	Event-related fMRI with visual stimulus (luminous stimulations at four intensities)	Wider photoresponsive area in the visual cortex in response to light, as well as hyperexcitability of the visual cortex respect to controls
Denuelle et al. (27)	8 MwoA, episodic	Ictal, post-ictal, and post treatment; spontaneous attacks	${\text{H}}_{2}^{15}$O PET with visual stimulus (luminous stimulations at increasing intensities to induce photophobia)	Increased activation in the visual cortex of migraineurs respect to controls, both during migraine attacks with photophobia and following headache relief with sumatriptan. Hyperexcitability was not present in the interictal phase
Huang et al. (28)	7 MwA, 4 MwoA episodic	Interictal	Event-related fMRI with visual stimulus (striped patterns)	Increased activation in visual cortex of migraineurs respect to controls
Descamps et al. (29)	21 MwoA episodic	Interictal	Event-related fMRI with visual stimulus (faces, short interstimulus intervals)	Repetitive visual stimuli in migraine showed an altered hemodynamic refractory response respect to controls, possibly confirming lack of interictal habituation
Datta et al. (30)	25 MwA, 25 MwoA	Interictal	Event-related fMRI with visual stimulus (checkerboard on/off pattern every 15 s)	Increased BOLD response to visual stimulation in V1 and LGN in MwA patients compared to both MwoA and controls
Hougaard et al. (31)	20 MwA episodic	Interictal	Event-related fMRI with visual stimulus (dartboard on/off pattern every 18 s)	Increased BOLD response to visual stimulation in downstream visual network areas (inferior frontal gyrus, superior parietal lobule, intraparietal sulcus, and inferior parietal lobule) of symptomatic aura hemispheres compared to controls
Griebe et al. (32)	18 MwA episodic	Interictal	Event-related fMRI with visual stimulus (optokinetic drum with colored figures)	Increased activation in visual motion perception areas (bilateral V5 complex and left area V3) as well as cuneus and precuneus
Maniyar et al. (33)	10 MwoA episodic	Ictal premonitory phase; induced attacks with GTN	${\text{H}}_{2}^{15}$O PET	Increased activation of cuneus (BA18, part of the extrastriate visual cortex) and right precentral gyrus (BA4) in patients with photophobia in the premonitory phase vs. baseline phase, respect to patients without photophobia
Niddam et al. (34)	26 MwA, 26 MwoA	Interictal	Resting-state fMRI (seed based; ROIs in salience network and dorsal attention network)	Decreased connectivity between the anterior insula and extrastriate areas (including V3A) in MwA compared to both MwoA and controls. The reduced connectivity correlated with headache severity
Tedeschi et al. (35)	20 MwA, 20 MwoA	Interictal	Resting-state fMRI (ICA)	Increased functional connectivity in the right lingual gyrus (within the resting-state visual network) in migraine aura patients, respect to migraine without aura and controls
Amin et al. (36)	16 MwoA	Interictal and ictal; induced attacks with PACAP38	Resting-state fMRI (seed based; ROIs in salience network, default mode network, and sensorimotor network)	Decreased connectivity in the sensorimotor network with the left visual cortex. Increased connectivity in the DMN with the visual cortices
Coppola et al. (37)	18 MwoA	Interictal	Resting-state fMRI (ICA)	Decreased connectivity between the default mode network and the visuospatial system
Hougaard et al. (38)	16 MwA	Interictal, ictal during aura	Resting-state fMRI (ICA + seed based; ROIs in cortical visual areas and areas of pain)	Increased functional connectivity between V5 and the ipsilateral middle frontal gyrus of the hemisphere contralateral to the perceived visual aura symptoms, following visual aura attack
Faragó et al. (39)	18 MwA, 35 MwoA	Interictal	Resting-state fMRI (ICA)	Increased amplitude of resting activity fluctuation in the lateral visual network in MwA patients respect to MwoA and controls
Arngrim et al. (40)	5 MwA	Interictal, ictal during aura; induced attacks with hypoxia, sham hypoxia, or physical exercise	Event-related fMRI with visual stimulus (dartboard on/off pattern every 18 s)	Reduced BOLD response in patients reporting scotoma and increased response in patients with positive aura symptoms. Bi-hemispherical BOLD changes in patients with bilateral visual symptoms
Lisicki et al. (41)	20 MwoA	Interictal	[18F]-FDG PET (with VEPs)	Increased neuronal activation-to-resting glucose uptake ratio in the visual cortex in patients
Lisicki et al. (42)	19 MwoA	Interictal	Resting-state fMRI (seed based)	Increased functional anti-correlations between the right temporo-parietal junction and the visual cortex in patients
Russo et al. (43)	17 MwA, 18 MwoA	Interictal	Event-related fMRI with noxious trigeminal heat stimulation	Increased activation of visual network (lingual gyrus, inferior parietal lobule, inferior frontal gyrus, and medial frontal gyrus) and midline-inferior cerebellum in patients with MwA compared to healthy controls and MwoA
Arngrim et al. (44)	15 MwA	Interictal, during hypoxia	Event-related fMRI with visual stimulus (dartboard on/off pattern every 18 s)	Greater hypoxia-induced decrease in BOLD following visual stimulation in visual areas V1, V2, V3, V4

Visual stimulation is capable of triggering migraine attacks, and this has shown to involve brainstem structures, in particular the red nucleus and substantia nigra (23). Furthermore, MwoA and MwA have been repeatedly associated with increased BOLD response in the primary visual cortex and higher-order visual areas, both during the interictal period (24, 25) and during visually triggered attacks (23).

Migraineurs, both with and without aura, show a more extensive photoresponsive area in the visual cortex in response to light (26) as well as a general increased response to visual stimuli (28). These patients also display a lack of interictal habituation for repetitive visual stimulation (29) in event-related fMRI studies in between attacks.

Spontaneous migraine attacks have also been associated with increased activity in the visual cortex with ${\text{H}}_{2}^{15}$O PET (27) in response to increasing intensities of light stimulation used to induce photophobia.

In an fMRI study investigating the same MwoA patient daily over the course of 30 days, a bilateral visual cortex activation (specifically Brodmann areas 17 and 18) was found in the 24 hours prior to attack onset, as well as in response to trigeminal nociceptive stimulation during the postictal phase. Interestingly, the same area showed significant deactivation during attacks compared with the interictal phase. These results indicate either an increased visual and nociceptive integration in the build-up of the migraine attack, which in turn reverts during the actual attack, or an increased activation of the visual cortex at baseline in migraineurs, whom therefore lack a normal occipital response during pain (45).

Functional connectivity (fc) is also altered within the visual network in MwoA. In a resting-state fMRI study using PACAP38 to induce attacks, decreased fc was found between the sensorimotor network and the left visual cortex, while conversely, increased connectivity was found between the default mode network (DMN) and the visual cortices bilaterally (36). It is possible that part of these BOLD signal changes were due to PACAP38 itself, given that it is a potent vasodilator, however, its effect on intracerebral arteries seems limited (46).

Another study found interictal fc reduction between the DMN and the visuo-spatial system in episodic migraineurs without aura in-between spontaneous attacks (37), whereas a more recent connectivity analysis in migraineurs without aura showed increased functional anti-correlation between the right temporo-parietal junction and the bilateral visual cortex (42).

Finally, a combined visual evoked potentials (VEPs) and [^18^F]-FDG PET study in interictal migraineurs without aura showed significantly reduced glucose uptake in the left BAs 19, 18, and 7, in patients. This results was present when regressing for the VEP area under the curve, thus suggesting an activity-induced rupture of cerebral metabolic homeostasis in migraine (41).

### Structural Alterations of the Visual Network

Several imaging studies have shown changes in cerebral gray matter (GM) and white matter (WM) volume in patients with migraine (Table 2).

**Table 2:** Main structural neuroimaging studies showing alterations of the visual network in migraine with (MwA) and without aura (MwoA).

**References**	**Patient cohort**	**Methodology**	**Main results**
Granziera et al. (47)	12 MwA 12 MwoA	DTI, cortical thickness	Increased cortical thickness in V3A and V5 in migraineurs respect to controls. Reduced fractional anisotropy in V3A and LGN in migraineurs
Rocca et al. (48)	7 MwA 8 MwoA	DTI	Altered tractography in optic radiations of migraineurs with visual aura respect to controls and patients without aura
Zhang et al. (49)	32 MwoA	VBM, DTI, SBM	Increased GM volume in the lingual gyrus, fusiform gyrus, and parahippocampal gyrus in patients respect to controls. Increased cortical thickness and gyrification index in lateral occipital cortex in patients
Coppola et al. (50)	20 MwoA, chronic	VBM	Decreased GM volume in left V1/V2 in patients respect to controls
Palm-Meinders et al. (51)	52 MwA 32 MwoA	VBM	Decreased GM volume in V3 and V5 in migraineurs respect to controls. V5 changes correlated with disease activity
Gaist et al. (52)	166 MwA	Cortical thickness	Increased cortical thickness in areas V2 and V3A in migraineurs with visual aura
Lisicki et al. (53)	20 MwoA	VBM	No differences in GM volume in patients respect to controls; positive correlation between GM volume in BA 17 and mean VEP amplitude

A cortical thickness and DTI study in 12 MwA and an equal number of MwoA patients showed an increase in the thickness of motion-processing areas V5 and V3A area in migraineurs respect to controls, accompanied by reduced fractional anisotropy in the WM subjacent to V3A as well as the lateral geniculate nucleus (LGN) (47). Another DTI study showed tractography alterations in the optic radiations of seven migraineurs with visual auras compared to healthy controls and migraineurs without visual aura (48).

Zhang et al. combined voxel-based morphometry (VBM), SBM, and DTI to investigate structural alterations in 32 MwoA patients compared to healthy controls. They found that migraineurs had increased GM volume in an area encompassing the lingual, fusiform, and parahippocampal gyri. Further, cortical thickness in the lateral occipital cortex and gyrification index in the right lateral occipital cortex were significantly increased in migraineurs. No changes in white matter microstructure using DTI were found in this study (49).

Slightly contradicting these results, Coppola et al. analyzed 20 patients with chronic MwoA and found decreased GM volume in the left primary occipital cortex and visual association areas (corresponding to Brodmann areas 17 and 18) with respect to healthy volunteers. It should be noted that these results only survived cluster-wise multiple comparisons correction at a more lenient cluster-forming threshold than normally adopted (50).

A larger study on 84 migraineurs both with (*n* = 52) and without (*n* = 32) aura showed a decrease in GM volume of visual areas V3 and V5 (Brodmann area 19) in patients, compared to controls. A *post-hoc* analysis showed that changes in V5 were more pronounced in migraineurs with an “active” disease (51).

In an elegant study comparing females with MwA to their unaffected twins and unrelated controls, Gaist et al. assessed the cortical thickness of V1, V2, V3A, and V5 areas, finding an increased thickness of areas V2 and V3A in the patient group (52). This alteration was not associated with clinical parameters such as disease activity or aura attack frequency, leading the authors to hypothesize that the morphometric changes represented an inherent trait of migraine with visual aura.

A recent study combining VBM and VEP by Lisicki et al. found no global differences in gray matter volume of migraine patients respect to controls. There was, however, a significant correlation in migraineurs between VEP amplitude and GM volume within the visual cortex, among other regions (53).

The opposing findings of increased and decreased gray matter volumes within the motion network in migraineurs are difficult to interpret. Variations within technical acquisition or image processing could be a relevant cause. Another important element could be the differences among study populations. Some authors investigated predominantly episodic (47, 49) while others exclusively chronic (50) migraine; others did not distinguish between the two (51, 52). Given that volumetric differences in area V5 and the cerebellum were associated with attack frequency (51) and acute medication intake, respectively (50), this is an aspect that certainly needs to be taken into account in the planning of future studies.

### Spectroscopy Investigations

The use of MRS has increased the already expanding knowledge on visual cortex activation in migraine, by studying *in-vivo* neuronal metabolism. ^1^H-magnetic resonance in particular allows to measure concentration of N-acetylaspartate (NAA), creatinine (Cr), glutamate (Glx), GABA, and lactate. Several studies performed with this technique in migraineurs—mostly with visual aura—have shown alterations of the visual system.

One paper investigating visual cortex metabolism in MwA, MwoA, and controls subject to visual stimuli, showed that photic stimulation caused a more sustained decrease of NAA and concomitant increase in lactate in MwA patients respects to the other groups, which the authors argue could highlighting potential abnormal mitochondrial function in aura subjects (54). This dysfunction was confirmed by Sandor et al., who studied visual cortex lactate changes in MwA following prolonged visual stimulation, and found that compared to controls or subjects with sensory or motor aura, patients with visual aura displayed abnormally elevated lactate levels, even at rest (55).

With simultaneous transcranial direct current stimulation (tDCS), VEP recording and spectroscopy in MwA patients, Siniatchkin et al. were able to show that occipital areas in migraineurs are characterized by altered homeostasis and cortical information processing (56). In the healthy controls of the study, excitatory and inhibitory baseline tDCS, respectively, triggered either an increase or decrease in Glx/Cr ratio, which could be reversed by photic stimulation. Migraineurs, however, showed decreased Glx/Cr ratio in response to both types of tDCS stimulation, and importantly this did not to return to baseline in response to visual stimulus.

Another study in migraine with visual aura patients showed a 10% reduction in occipital cortex GABA concentrations respect to controls, as well as significant correlations between glutamate levels and BOLD response to visual stimulation that was not seen in controls. This suggested an altered excitation-inhibition coupling in MwA patients (57). Finally, a recent paper assessed the levels of visual cortex glutamate in both MwA and MwoA, finding higher Glx levels in migraineurs without aura compared to controls (58).

Overall, these studies suggest abnormal cortical processing of visual information and lack of habituation in between attacks in migraineurs, possibly due to an underlying metabolic dysfunction.

The picture that emerges from imaging across different modalities, is that of multiple functional, structural, and metabolic abnormalities affecting the visual network of migraineurs. The motion network in particular seems to be most significantly affected. This is true both for the extensive functional alterations found in the primary visual processing areas of V1/V2, which have specific sub-compartments involved in motion detection, as well as for the structural differences that multiple studies have uncovered in areas V3A and V5.

## Migraine With Aura

By far the most common clinical manifestation of aura is represented by visual symptoms that are prototypically characterized by an arc-shaped scintillating scotoma (59), although a high variability in symptomatology across and within patients has been recorded (60). The phenomenon of migraine aura has interested clinicians and researchers since its earliest descriptions. In recent decades, the mechanism of aura has become better understood, particularly thanks to seminal neuroimaging studies (61).

The most likely electrophysiological event underlying aura is cortical spreading depression (CSD), first described by Leão in the 1940s (62) and characterized by a wave of neuronal hyperexcitation followed by a sustained depression, traveling at a rate of 2–6 mm/min.

The most prominent evidence linking aura to CSD has come from a study involving the near-continuous recording of a patient with two aura attacks through the use of functional MRI (22). This showed that retinotopic progression of visual aura symptoms was congruently linked to an increase and successive decrease of BOLD signal, starting in cortical area V3A of the extrastriate cortex and progressing contiguously over the occipital cortex. Area V3A is linked to both motion processing and luminance contrast; it further has a retinotopic representation of the opposite hemifield (63). A more recent study in five MwA patients confirmed this link between BOLD changes and aura symptoms, and even showed that clinical heterogeneity in aura—such as prominence of positive or negative symptoms—corresponds to differences in BOLD signaling in the visual cortex. This paper in fact showed that the typical scotoma is associated with a reduced BOLD response likely caused by the depression in neural activity linked to CSD, whereas positive symptoms are linked to an increase in BOLD (40).

One debate regarding migraine aura has centered on the question of whether it represents a separate entity with respect to MwoA, and whether migraine pain can actually be caused by CSD itself.

A theory linking migraine pathogenesis to “silent CSD attacks” largely relies on animal studies showing that CSD can activate trigeminovascular neurons (64). The study by Cao et al. failed to find evidence in support of this hypothesis however, showing rather that activation of substantia nigra and red nucleus anticipates occipital cortex changes in spontaneous and visually triggered migraine with aura attacks (23). A more recent study demonstrated that, following visual aura attacks, there is increased connectivity between the pons and the somatosensory cortex and between V5 and the ipsilateral lower middle frontal gyrus; however, it found no differences in connectivity between visual cortex and pain areas (38).

Taken together, these studies seem to suggest that brainstem mechanisms contributed to the generation of pain attacks in both MwA and MwoA, and that involvement of the cortex in aura is a subsequent, parallel phenomenon.

Altered excitability of the visual pathways certainly plays a prominent role in the pathophysiology of MwA. Several neuroimaging studies have shown hyperexcitability of both primary and secondary visual cortices, even outside of the attacks.

Vincent et al. first showed that, following visual activation simulating the typical “zigzag lines” percept of aura, patients showed enhanced interictal reactivity of the extrastriate cortex respect to healthy subjects (24). MwA patients also show a stronger BOLD activation in the primary visual cortex and lateral geniculate nuclei compared to both healthy volunteers and MwoA patients, even when matched for levels of visual discomfort (30). Further, in the affected hemisphere of migraineurs with aura, response to visual pattern stimulation has shown to be increased in several downstream areas of the visual network involved in perception of motion, oculomotor control, visual attention and spatial memory (31). This is also seen following more complex forms of optokinetic stimulation (32). Finally, in response to an hypoxia challenge, patients with aura exhibit a greater decrease in BOLD signaling following visual stimulation, possibly due to higher blood oxygen extraction secondary to increased cortical excitability or to an abnormal vascular response (44).

Neuroimaging studies have also shown altered functional connectivity in MwA. Niddam et al. showed that MwA, compared to MwoA, had weaker fc between anterior insula and V3A, suggesting abnormal connections between the limbic and visual systems in aura (34). Faragó et al. found that MwA subjects present resting-state alterations within the lateral visual network respect to controls and MWoA, with increased amplitudes of resting BOLD fluctuations in the cingulate cortex, superior parietal lobule, cerebellum and bilateral frontal regions (39). Tedeschi et al. compared resting-state connectivity in the ictal phase of MwA vs. MwoA and controls, finding stronger fc within the visual network, particularly the extrastriate regions within the lingual gyrus (35). Interestingly, this resting-brain alteration was not limited to the aura phenomenon and was not correlated with clinical parameters or morphological differences, leading the authors to hypothesize that increased extrastriate cortical connectivity could represent a functional biomarker of MwA, differentiating it from MwoA and the non-migrainous brain. The same group has also recently demonstrated an abnormal response to trigeminal nociceptive stimulation in the lingual gyrus, inferior parietal lobule and cerebellum of MwA patients. This confirms the involvement of areas of higher visual processing in MwA, and possibly shows that functional integration between visual and trigeminal pain networks could represent a key pathophysiological mechanisms underlying migraine with aura (43).

Overall, these studies show that both lower and higher visual processing is impaired in aura patients, ictally and interictally. The visual cortices generally present hyperexcitability in response to visual stimulus in migraine with aura. Further, functional connectivity seems to be increased within the visual network and conversely decreased between the visual network and other key brain structures in MwA. Even if these characteristic are not limited to MwA, they certainly seem to be more prominent in this subpopulation.

## Photophobia

Typically, light can either exacerbate ongoing migraine pain (photic allodynia), or it can be perceived as very bright or uncomfortable (photic hypersensitivity). Photophobia and migraine pain are directly correlated, with light stimuli causing lower thresholds to pain in trigeminal innervated locations in migraineurs (65, 66), and painful trigeminal stimulation leading to decreased visual discomfort thresholds (5). Importantly, photophobia prevalence appears to be independent of migraine aura (65). Nonetheless photic sensitivity is a key aspect of migraine biology, representing not only a prominent feature of the attack (67), but commonly present in the premonitory (68) and interictal phases also (69).

Photophobia is also frequently experienced as part of the visual snow syndrome (19), underlying the important pathophysiological link between these two conditions.

Several studies have investigated the mechanism of photophobia in migraine, with one prominent paper that identified a pathway through which photic signals from the retina converge on nociceptive pathways mediating migraine pain (70), likely explaining the exacerbation of headache by light.

In a ${\text{H}}_{2}^{15}$O PET study, Boulloche et al. showed that in response to light stimulation migraineurs had increased activation of visual network areas—specifically the cuneus, lingual gyrus and posterior cingulate cortex—respect to controls (6). Furthermore, this increased activation was potentiated by trigeminal pain, demonstrating a close interrelation between light perception and the trigeminal nociceptive pathway. The same group then directly investigated ictal photophobia in spontaneous attacks of MwoA, finding that light sensitivity was linked to an increased activation in the visual cortex present during the attack, involving the areas of the lingual gyrus and the cuneus (27). With the same technique, Maniyar et al. studied photic sensitivity in premonitory phase of glyceryl trinitrate (GTN) induced attacks of MwoA and found that premonitory photic hypersensitivity is linked to activation of extrastriate visual cortex, specifically Brodmann areas 18 and 4 (33).

In a functional MRI study on interictal chronic migraineurs, authors found an altered connectivity between the anterior insula and pulvinar of patients with migraine, which could explain, at least in part, the abnormal perception of visual stimuli as painful (71). The pulvinar is relevant in selecting salient visual stimuli (72) and has a direct role in the integration between trigeminal pain and visual inputs (70) through a pathway involving the optic nerves and dura-sensitive spinal trigeminal nucleus neurons (73).

These studies suggest that migrainous photophobia is characterized by diffuse associative visual cortex abnormalities, and that these are possibly linked to abnormal sensory processing in thalamic structures, particularly the pulvinar.

## Visual Snow

Visual snow is a neurological disorder characterized by a continuous visual disturbance that takes the form of uncountable tiny flickering dots covering the whole visual field (74). This static disturbance is often referred to as “snow;” it is typically black and white but can also be colored, flashing, or transparent. In the more complex visual snow syndrome, patients experience several other visual symptoms, that can be of neurological origin—such as palinopsia, photophobia, and nyctalopia—or originate directly from the optic apparatus. The latter are called “entoptic phenomena” and manifest in the syndrome with various combinations of blue field entoptic phenomenon (BFEP), floaters, self-light of the eye, and/or spontaneous photopsia (19).

Even if visual snow represents an entity distinct from both migraine without aura and typical migraine aura, comorbid migraine is present in up to 80% of visual snow cases, significantly complicating its phenotype (75–77). In particular, VS patients who have comorbid migraine present an increased chance of having non-entoptic visual symptoms. Further, some cases of VS has been reported to start with an aura episode (18).

To date there has been only one neuroimaging investigation on VS syndrome, and this was an [^18^F]-FDG PET study performed on 17 patients (75). This study demonstrated that patients with VS exhibit increased brain metabolism in the area of the right lingual gyrus compared to healthy volunteers. The distribution of hypermetabolism was very similar to the area also shown to be directly linked to ictal photophobia in migraine (27), further supporting the hypothesis of a pathophysiological overlap between the conditions.

### Toward a Model for Visual Snow

The lack of recognition of the visual snow condition, which was only characterized very recently, has posed a challenge to understanding the biology underlying this disorder. The consistency of the clinical description offered by affected patients allows to hypothesize a common, general, pathophysiological mechanism, although it is also possible for different aspects to be more relevant in-between subjects.

We here outline possible theories on the visual snow pathogenesis, proceeding anatomically from the periphery onto higher areas of visual processing, and hypothesizing on a common biology underlying the condition by analyzing the different features that characterize it.

The first, most obvious, explanation for VS is that it is directly or indirectly triggered by an eye disease. Several ophthalmologic cases can present with clinical features of “static” similar to visual snow. The authors themselves (FP/PJG) received an unpublished report of VS in a subject diagnosed with X-linked Retinitis Pigmentosa. Indeed a de-afferentation syndrome, in which even a temporary alteration in retinal firing causes a dissociation between peripheral sensory input and central visual perception, would explain the similarity of visual snow to tinnitus, a highly common comorbidity (74) and in some respects the auditory counterpart of visual snow. A similar mechanism is also present in the classic hallucinatory condition of Charles-Bonnet syndrome (CBS), where progressive loss of visual function causes hypo-connectivity from the visual periphery to the brain and gives rise to hallucinations (78). It is also tempting to explain the associated entoptic phenomena of VS syndrome as something arising plainly from the eye, as they are indeed typically described in ophthalmic disorders (79) and can even be present in healthy individuals as a consequence of floating strands of vitreous or white blood cells within the microvasculature stimulating retinal neurons (80–82).

The main counter-argument to interpreting VS as a purely eye phenomenon however, lies primarily in the absence of ophthalmic disorders, a required criterion for the diagnosis of VS (19), and also in the normality of basic eye electrophysiology, such as ERG or VEPs, reported in VS cohorts (74, 76). This does not exclude that perhaps some cases of visual snow might be caused by eye disorders. In this respect it is interesting to recall that in certain examples, CBS hallucinations are characterized by simple flashes, dots of light, or palinopsia, an important feature of VS syndrome (83). More case studies are clearly needed to further elucidate the interaction of eye disorders and visual snow-like phenomena.

A second theory on VS pathophysiology involves a direct thalamic dysfunction. In a process known as thalamo-cortical dysrhythmia, a dissociation exists between sensory inputs from the thalamus and its projections to the cortex. This mechanism was first described by Llinas in tinnitus (84), and is characterized by an increase in unusual, large-scale and coherent thalamo-cortical low-frequency oscillations. These delta and theta oscillations are likely caused by a switch from tonic to high-frequency thalamic bursting—due to protracted cell hyperpolarization—and ultimately determine a disintegration of sensory perception at the cortical level. It is certainly possible to hypothesize a role for thalamo-cortical dysrhythmia in visual snow. Potentially, an underlying homeostatic imbalance of visual pathways, either from altered retinal activity or genetic predisposition, could cause a disinhibition of projections from the posterior thalamus to primary and secondary visual cortices and parietal cortex as well—explaining palinopsia and a continuous perception of movement—thus affecting normal visual perception (76).

Interestingly, the thalamo-cortical dysrhythmia hypothesis also seems to be relevant for migraine pathophysiology (85), where a functional disconnection of the thalamus is thought to be contributing to the abnormal habituation deficit repeatedly observed (86).

In a more simplistic view, the thalamus could be responsible for VS symptoms through a localized increase in activity of the LGN or the pulvinar. The pulvinar is part of the “thalamic matrix” and projects diffusely to the cortex, playing a significant role in cognition and attentive stimulus processing (87). Recent studies have confirmed that the pulvinar can facilitate attention-related communication across widespread neuronal networks including higher-order sensory cortices (88) and, as mentioned before, it has a clear role in photophobia. In the future, neuroimaging studies focused on these nuclei will help clear the role of thalamic dysfunction in visual snow.

A third option could be to hypothesize VS as a purely cortical phenomenon. In visual hallucinatory syndromes, the percept of hallucinations has been shown to correspond to a dysfunction in the cortical area where that particular perception is represented (89). If the “cortical dysfunction theory” were true, we should therefore expect altered brain structure, compensatory neuroplasticity or functional activity to be constrained to visual association/motion areas. It is known that topological visual disorders caused by hyper-function in V1/V2 areas can present with hallucinations similar to visual snow (90). Further, a recent case of sporadic Creutzfeldt-Jakob disease presenting with features of visual snow has been reported in the literature (91). These cases are, however, exceptional and they would certainly not explain most cases of VS in which no gross central nervous system abnormalities are found.

A more complex explanation of the role of the cortex could involve a widespread dysfunction of higher-order visual processing areas, particularly the extrastriate cortex. Certainly the cited PET study, showing increased metabolic activity in the lingual gyrus, points to this (75). There have also been important neurophysiological (92–94) and behavioral (95) studies demonstrating an altered processing and dishabituation in the visual network of the VS brain.

The dorsal visual network, involved in processing visual motion, is likely to play a role in a condition characterized by the perception of constantly moving objects. The motion network is part of what has now been renamed as the “how-pathway” (96) and spreads from V1 dorsally to the parietal lobe, involving visual motion area V5 located in the temporo-parietal-occipital junction (97).

Finally, an altered connection between visual networks and other brain networks involved in salience, cognition and interoception is possible in a disorder like VS. Vision is a dynamic, active process in which top-down influences are seen at all stages of the visual hierarchy—with the exception of the retina—and control various functional properties of vision, particularly attention (98). We can hypothesize that visual snow may be characterized by a general altered excitability and connectivity of the visual network with either the salience and/or DMNs, which typically exert top-down influence on the visual cortex, = or the dorsal and ventral attentional networks, which have been abundantly implicated in theories of visual hallucinations (99, 100).

**Figure 1:**
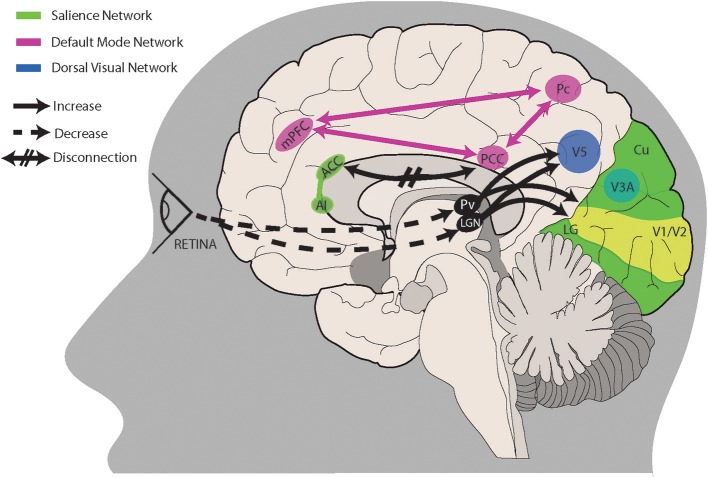
A proposed model for visual snow pathophysiology. Altered peripheral visual stimulation or a form of genetic predisposition could induce dysrhythmic connections between thalamic structures and cortical visual areas. The lateral geniculate nucleus (LGN) and pulvinar (Pv) in particular are directly connected to motion area V5 and the lingual gyrus (LG). Relevant to visual snow biology is the motion processing network, which is composed of areas within the primary visual cortex (V1/V2), area V3A within the cuneus (Cu), area V5 located ventrolaterally among the lateral occipital sulcus and inferior temporal sulcus, and Brodmann area 7 in the precuneus (Pc). Structures pertaining to the default mode network (PCC, posterior cingulate cortex; Pc; mPFC, middle prefrontal cortex) and/or the salience network (AI, anterior insula; ACC, anterior cingulate cortex) are involved in salience and interoception. Disruption of these networks, possibly through altered connectivity between cortical areas, could also play a role in visual snow pathophysiology. See main text for a more in-depth explanation.

The final, overarching framework that we propose for visual snow encompasses the three aforementioned hypotheses. If a combination of peripheral, subcortical and cortical dysfunctions were all at play, either in different subjects or in different moments of the natural disease history, this would explain not only the main symptom of the snow common to all patients, but also the variety of symptoms characterizing the VS syndrome. Similarly to a model that has been used to explain tinnitus and is potentially involved in chronic pain as well (101), we could imagine that subcortical spontaneous activity normally ignored and considered as erroneous by the brain in normal conditions, might for various reasons increase in salience and be considered as the default visual perception, particularly if the hierarchical sensory processing networks in the brain do not correct this faulty perception. This model would certainly explain the continuous background perception of the simple static or snow, but also the more complex phenomena typical of the syndrome: palinopsia, entoptic phenomena, photophobia and even nyctalopia, which could in fact simply represent an increased perception of the “noise” when no other stimulus is present. Figure 1 summarizes the salient aspects of this theory, showing the most important brain structures and connections likely involved in visual snow pathophysiology. Neuroimaging studies will be particularly useful in the future to determine the strength of this reasoning, as well as the role of the different mechanisms in VS biology.

A continuous dysfunction of large-scale visual processing networks, in particular of the motion network, through this or other mechanisms in visual snow possibly constitutes a link to its “cousin” condition of migraine, in which manifestations of altered visual processing, although not predominant, constitute an important aspect of a disease characterized by generalized alterations of sensory processing.

## Conclusions

In summary, modern neuroimaging has allowed to detect several functional, structural and metabolic changes affecting multiple elements of the visual network in migraineurs, both with and without aura. These abnormalities help explain some of the key features of the condition, such as abnormal sensory processing, photophobia and the aura phenomenon, and further link it to the growingly recognized neurological syndrome of visual snow. In this condition, which is likely on a similar pathophysiological spectrum as migraine, multiple elements (i.e., cortical hypermetabolism, thalamo-cortical dysrhythmia, brain network dysfunctions) could be at play in the generation of a persistent visual illusion.

## Author Contributions

FP wrote the first draft of the manuscript. DF and OO'D revised the initial drafts and gave scientific contribution. PG edited the final version of the manuscript.

### Conflict of Interest

The authors declare that the research was conducted in the absence of any commercial or financial relationships that could be construed as a potential conflict of interest.
